# Genetic variability of Shiga toxin-producing *Escherichia coli* strains isolated from Paraguayan cattle

**DOI:** 10.1128/spectrum.00596-25

**Published:** 2025-08-14

**Authors:** Claudia Salinas, Fátima Rodriguez, Adrián Muñoz-Barrera, José Miguel Lorenzo Salazar, Rafaela González-Montelongo, Carlos Flores, Rosa Guillén

**Affiliations:** 1 Departamento de Microbiología, Instituto de Investigaciones en Ciencias de la Salud, Universidad Nacional de Asunción 202653 , San Lorenzo, Paraguay; 2 Genomics Division, Instituto Tecnológico y de Energías Renovables S.A. (ITER) 601110, Santa Cruz de Tenerife, Spain; 3 Research Unit, Hospital Universitario Nuestra Señora de Candelaria, Instituto de Investigación Sanitaria de Canarias, Santa Cruz de Tenerife, Spain; 4 Centro de Investigación Biomédica en Red de Enfermedades Respiratorias (CIBERES), Instituto de Salud Carlos III 38176, Madrid, Spain; 5 Facultad de Ciencias de la Salud, Universidad Fernando Pessoa Canarias 465361, Las Palmas de Gran Canaria, Spain; University of Maryland at College Park, College Park, Maryland, USA

**Keywords:** *Escherichia coli*, Shiga toxin, cattle, Paraguay, whole-genome sequencing

## Abstract

*Escherichia coli* is a commensal of the intestinal microbiota; however, there are pathogenic strains associated with both intestinal and extraintestinal conditions. One of these pathotypes is Shiga toxin-producing *E. coli* (STEC), which can cause diarrhea, hemorrhagic colitis, and hemolytic uremic syndrome and has cattle as one of its main reservoirs. This study aimed to characterize genomes of STEC strains isolated from rectal swabs of cattle from ranches in the departments of San Pedro, Cordillera, Caaguazú, Paraguarí, and Villa Hayes in Paraguay. The previous characterization of the isolates and their classification as STEC were carried out by conventional PCR to identify the presence of the *stx1* and *stx2* genes. Here, whole genome sequencing was performed on 40 strains in total (38 STEC and 2 non-STEC) using the Illumina MiSeq platform. The presence of 27 different virulence profiles and 34 different serotypes was detected. Regarding antibiotic resistance, the *fosA7* gene related to fosfomycin resistance was detected in 10% of the strains (*n* = 4), and the *sitABCD* gene related to hydrogen peroxide resistance in 2.5% (*n* = 1). Twenty-nine sequence types were detected, with ST58 (*n* = 3) and ST11729 (*n* = 3) being the most frequent. Most of the strains belong to phylogroup B1. This is the first report on the genetic variability of STEC strains isolated from cattle in Paraguay. Given the importance of livestock in the country’s economy, epidemiological surveillance of pathogenic strains should continue to be carried out.

**IMPORTANCE:**

Shiga toxin-producing *Escherichia coli* (STEC) can cause serious foodborne illnesses in humans. Cattle are a natural reservoir of STEC, and transmission to humans occurs through consuming contaminated food, direct contact between humans and animals, and from person to person through the fecal-oral route. This study analyzed the genetic variability of STEC strains from cattle in Paraguay. The findings highlight the genetic diversity of STEC in Paraguay and emphasize the need for continued monitoring, as livestock play a key role in the country's economy. Applying genomic surveillance can help improve food safety, prevent outbreaks, and protect public health.

## INTRODUCTION

Paraguay ranks ninth among the countries with the highest volume of beef exports worldwide, with the livestock sector accounting for 12% of the country’s gross domestic product. The country is divided into 17 departments: 14 in the Eastern Region, with 52% of the cattle farms, and 3 in the Western Region of the Chaco, with 48% of the cattle farms of the country (1). In addition to its significant export volume, Paraguay has a high *per capita* beef consumption (2). Due to the prominence of the livestock sector, the constant surveillance of foodborne pathogens associated with meat production is essential not only from an economic point of view but also from a public health point of view (3, 4).

*Escherichia coli* is a gram-negative bacterium that is part of the intestinal microbiota of animals; however, although most strains are non-pathogenic, some can cause intestinal and extraintestinal infections, affecting the respiratory and urinary tracts, bloodstream, skin, and soft tissues. Pathogenic strains that cause enteric diseases are called diarrheagenic and are classified into six main pathotypes, including enterotoxigenic *E. coli*, enteropathogenic *E. coli*, enteroinvasive *E. coli*, enteroaggregative *E. coli*, diffusely adherent *E. coli*, and Shiga toxin-producing *E. coli* (STEC) (5, 6). In recent years, an increasing number of hybrid or heterogeneous strains have been described, carrying virulence genes from more than one pathotype. These strains challenge traditional classification schemes and may exhibit enhanced pathogenic potential or broader host adaptation (7).

STEC infection can lead to several severe conditions, including hemolytic uremic syndrome. These bacteria are known for producing Shiga toxins (Stx1 and Stx2). These toxins not only induce cell death by inhibiting protein synthesis, but they can also trigger systemic inflammatory responses through other pathways. This can result in multiple organ failure, increasing both the morbidity and mortality associated with infections caused by these bacteria (8). In addition, its treatment becomes complicated because administering antibiotics at sublethal doses can increase the production of the toxin (9). While Shiga toxin is their main characteristic, the pathogenic capacity of the strains is determined by the presence of other virulence factors (10).

One of the main ways to characterize STEC strains is through serotyping the O and H antigens. Serotype O157:H7 is best known to cause human infections; however, other serogroups have caused outbreaks and severe cases (11).

Meat is the type of food most commonly associated with STEC outbreaks (12), and this is because cattle are one of the main reservoirs of these strains. If these animals' feces are used as fertilizer, they can contaminate water and soil, contaminating other types of food. Different forms of transmission include cross-contamination during food preparation, direct human-animal contact, and person-to-person by the fecal-oral route (13).

Due to the lack of genomic data related to these pathogens in the country, this study aimed to perform the first whole-genome sequencing (WGS)-based molecular characterization of STEC isolates obtained from cattle ranches in five departments of Paraguay. This characterization will help define the genetic diversity, virulence, and resistance profiles of circulating strains, which is essential to assess public health risks and surveillance strategies.

## MATERIALS AND METHODS

### Sample collection

We analyzed bacterial isolates from fecal samples collected from 241 healthy cattle raised on farms located in five departments of Paraguay. Of these animals, 143 were from the Eastern Region (San Pedro, Cordillera, Caaguazú, and Paraguarí), and 98 were from the Western Region (Villa Hayes). In 2013, samples were collected from two farms: one in Villa Hayes, from which 98 samples were obtained, and one in Paraguarí, with 99 samples collected. In 2016, an additional 44 samples were obtained from 11 different farms located in the departments of Cordillera, San Pedro, Paraguarí, and Caaguazú, with four randomly selected animals sampled per farm. All cattle were between 4 and 8 months old, and animals receiving antibiotic treatment were excluded from the study. Fecal samples were collected by swabbing the external anal area of each animal using sterile cotton swabs with Stuart transport medium (Copan, USA).

### Sample processing

Swabs were streaked onto McConkey agar and incubated at 37°C for 24 hours. The resulting confluent growth zone was considered representative of the bacterial population present in each sample. Two aliquots were obtained from this zone: one was used to extract total DNA for initial screening, and the other was cryopreserved at −80°C. Detection of *stx1* and *stx2* genes was performed on the pooled DNA using multiplex PCR, following the protocol described by Blanco et al. (14). Only samples that tested positive for *stx1* and/or *stx2* were processed further. For each positive sample, multiple cultures were made from the original confluence zone to isolate individual colonies. Up to 50 colonies per sample were screened to identify STEC-positive isolates. Colonies carrying *stx1* and/or *stx2* were subsequently subjected to PCR for detection of the 16S rRNA gene (15); and to biochemical tests, including triple sugar iron, lysine iron agar, sulfide indole motility, and Simmons citrate agar; to confirm species identity and to ensure consistency between molecular and traditional phenotypic approaches.

### Whole-genome sequencing and genotypic characterization

Thirty-eight STEC isolates were selected for WGS based on virulence profiles previously determined by conventional PCR. Also, two non-STEC isolates were sequenced. This served as a control, validating the general selection process carried out during the project and ensuring the reliability of our findings. The bacterial DNA was extracted using the Wizard Genomic DNA Purification Kit (Wizard Genomic, Promega, Madison, USA) following the manufacturer’s instructions. The libraries were prepared using the Nextera XT DNA Library Preparation Kit (Illumina Inc., California, USA). Sequencing was performed using the MiSeq sequencing platform (Illumina Inc., California, USA). The library was loaded at a concentration of 14 pM, and 1% Control PhiX was used as an internal control. The sequencing was conducted at the Institute of Technology and Renewable Energy (ITER, Tenerife, Spain).

BCL files were converted to demultiplexed FASTQ files using the bcl2fastq2. Quality control was performed with FastQC v0.74 to assess sequencing quality, read length, and total number of reads. The process of trimming and assembling the sequences and subsequent analysis was carried out using the Galaxy platform (16). Fastp v0.23.2 was used to improve the quality of the sequences (17). The *de novo* genome assembly was made using Unicycler v0.5.0 (18), and QUAST v5.2.0 was used to assess assembly quality (19). A summary report was obtained with assembly metrics such as total genome size, total number of contigs, largest contig size, and contig with a size greater than 1 kb, N50, and the GC content. Genomes were annotated with Prokka v1.14.6 (20). The ABRicate tool v1.0.1 of the Galaxy server was combined with the EcOH, Ecoli_VF, and ResFinder databases for the identification of serotype, virulence factor carrier profile, and genes related to antibiotic resistance, respectively. The sequence type was determined using the MLST tool v2.22.0. All analyses were performed using default parameters. We used the NG-CHM Builder: Cluster Matrix platform from the University of Texas v2.22.2 to generate the heatmap with the virulence factor profiles detected (21). The phylogroup of the isolates was determined using the ClermonTyping web tool (22).

The sequences of the phages present in the isolates were obtained using the PHASTEST tool (23), and the comparison of these sequences and the WGS with the reference sequences of the *stx1* (AB048237.1) and *stx2* (AF043627.1) genes was performed with BLASTN (NCBI). The verification of the absence of non-specific amplifications of the primers used for the screening PCR to detect the *stx1* and *stx2* genes was performed with the Primer Search tool v5.0.0 of the Galaxy server. The sub-typification of the *stx* genes was also performed using the same tool for the primers described by Scheutz et al. (24, 25). Based on the carrying of the different subtypes of toxins, the isolates were categorized according to their potential to cause severe disease in humans using FAO/WHO criteria (12).

Phylogenetic analyses were performed using Roary v3.13.0 (26) pipeline to generate the alignment of the core genome of the isolates. Then, FastTree v2.1.10 was used to generate a maximum-likelihood phylogenetic tree using the GTR+CAT evolutionary model (27). Nine isolates corresponding to STEC strains isolated in the Americas reported in previous studies were used to obtain a broader view of the genomic relationship between isolates. These included strains corresponding to serogroups O26, O45, O103, O111, O121, and O145 (the group known as the “big six”); O157, O74, and O22; and isolates from a study conducted in Brazil (28–30). The data for these reference strains (year of isolation, origin, and publication) are detailed in Table 1. The iTol v6.9 tool was used to visualize the tree and the data of the isolates (31).

**TABLE 1:** Reference strains used for phylogenetic analysis

Serotype	Reference number	Origin	Country	Year	Source
O26:H11	CFSAN066388	Cattle	Chile	2016	(29)
O103:H2	MOD1-EC5236	Human	Argentina	1999	(29)
O111:H8	IHSV7	Human	Uruguay	2017	(29)
O145:H25	IHSV50	Human	Uruguay	2016	(29)
O121:H19	GCA_005037715	Human	Canada	2003	(28)
O45:H2	GCA_005037845	Human	Canada	2005	(28)
O157:H7	EC-0015	Human	Paraguay	2009	(29)
O74:H42	B-12	Cattle	Chile	2018	(29)
O22:H8	CFSAN066306	Beef	Chile	2016	(29)
O8:H20	GCA_022405635.1	Beef	Brazil	2015	(30)
O8:H20	GCA_022359875.1	Beef	Brazil	2015	(30)
O83:H19	GCA_022359895.1	Beef	Brazil	2015	(30)
O153:H25	GCA_022359845.1	Beef	Brazil	2015	(30)

## RESULTS

### Concordance between STEC identification by PCR and WGS

Of the total of 241 rectal swabs collected, 211 (87.5%) tested positive in the initial screening for at least one of the *stx* genes. Among the 38 isolates characterized by WGS, which were initially classified as STEC by conventional PCR, 28 were confirmed as STEC based on the presence of at least one of the genes that code for the toxins in the WGS analysis. Of these 28 isolates, 12 contained the *stx1* gene, while 22 contained the *stx2* gene. The presence of both the subunits A and B was detected in all cases, except for strain PY14-1, in which only the presence of the *stx1* subunit B was detected, and for strain PY50-1, in which only the presence of the *stx2* subunit B was detected. Of these 28 isolates, 18 had the same profile as the one obtained with the PCR. The primer search tool was used to confirm that the differences in gene detection were not due to non-specific primer amplification. The results matched those obtained by conventional PCR, except for strains PY14-1 and PY50-1, which only contain one subunit. A comparison of *stx1* and *stx2* reference sequences against the whole genomes was performed, obtaining a proportion of coverage ≤60% (Table 2).

**TABLE 2:** Concordance between STEC identification by PCR and WGS^*b*^

Strain	ST	SG	PCR	WGS	*In silico* PCR	BLAST (%Cov; %ID)	Sub-typification
PY1-3	58	O65:H8	*–*	*–*	*–*		
PY2-3	394	O17:H18	*stx1*,* stx2*	*stx1*,* stx2*	*stx1*,* stx2*		*stx1a*,* stx2d*
PY2-4	11729	O74:H42	*stx1*,* stx2*	*stx1*,* stx2*	*stx1*,* stx2*		*stx1a*,* stx2c*
PY3-1	196	O8:H7	*stx2*	*stx2*	*stx2*		*stx2a*
PY3-2	162	O8:H19	*stx2*	*stx2*	*–*		*stx2a*
PY4-1	1423	O48:H7	*stx1*,* stx2*	*stx2*	*stx2*	*stx1 *(47%; 66%)	*stx2a*
PY4-3	442	O156:H21	*stx1*,* stx2*	*stx1*,* stx2*	*stx1*,* stx2*		*stx2c*
PY5-3	6353	O132:H18	*stx2*	*–*	*–*	*stx2 *(8%; 95%)	
PY5-4	906	O150:H8	*stx2*	*–*	*–*	stx2 (8%; 95%)	
PY6-3	3576	O8:H7	*stx1*,* stx2*	*stx1*	*stx1*	*stx2 *(39%; 67%)	*stx1a*
PY6-4	3234	O21:H8	*stx2*	*stx2*	*stx2*		*stx2a*
PY7-2	ND	O159:H21	*stx1*,* stx2*	*–*	*–*	*stx1 *(0%),* stx2 *(8%; 95%)	
PY7-3	10079	O129:H23	*stx1*,* stx2*	*–*	*–*	*stx1 *(0%),* stx2 *(4%; 95%)	
PY8-1	3692	O2:H45	*stx1*,* stx2*	*stx1*	*stx1*	*stx2 *(39%; 67%)	*stx1a*
PY8-2	ND	OgN12:H31	*stx2*	*–*	*–*	*stx2 *(5%; 77%)	
PY9-1	164	O27:H8	*stx1*,* stx2*	*–*	*–*	*stx1 *(0%),* stx2 *(5%; 77%)	
PY9-2	1248	O5:H21	*stx1*,* stx2*	*stx2*	*stx2*	*stx1 *(47%; 66%)	*stx2b*,* stx2c*
PY10-2	200	O128:H28	*–*	*–*	*–*		
PY10-4	1611	O125:H19	*stx1*,* stx2*	*–*	*–*	*stx1 *(0%),* stx2 *(5%; 77%)	
PY11-1	58	O49:H30	*stx1*,* stx2*	*–*	*–*	*stx1 *(0%),* stx2 *(5%; 77%)	
PY14-1	1423	O48:H7	*stx1*,* stx2*	*stx1*^*a*^,* stx2*	*stx2*		*stx2a*
PY25-1	297	O179:H8	*stx2*	*stx2*	*stx2*		*stx2d*
PY29-1	99	O96:H19	*stx2*	*stx2*	*stx2*		*stx2a*
PY34-1	1727	O20:H7	*stx2*	*stx2*	*stx2*		*stx2c*
PY42-1	11729	O74:H42	*stx1*,* stx2*	*stx1*,* stx2*	*stx1*,* stx2*		*stx1a*,* stx2c*
PY46-1	362	O7:H10	*stx1*,* stx2*	*stx1*	*stx1*	*stx2 *(39%; 67%)	*stx1a*
PY50-1	4145	O22:H16	*stx2*	*stx2* ^*a*^	*–*		*stx2b*,* stx2d*
PY53-1	297	O93:H46	*stx2*	*stx2*	*stx2*		*stx2d*
PY58-1	658	O185:H28	*stx1*,* stx2*	*stx1*,* stx2*	*stx1*,* stx2*		*stx1a*,* stx2a*
PY101-1	2520	OgN31:H49	*stx1*,* stx2*	*stx2*	*stx2*	*stx1 *(47%; 65%)	*stx2a*
PY110-1	348	O110:H28	*stx1*,* stx2*	*stx1*	*stx1*	*stx2 *(50%; 66%)	*stx1d*
PY119-1	162	O8:H19	*stx1*,* stx2*	*–*	*–*	*stx1 *(11%; 71%),* stx2 *(37%; 95%)	
PY175-1	388	O153:H41	*stx1*,* stx2*	*stx2*	*stx2*	*stx1 *(58%; 65%)	*stx2a*,* stx2b*
PY194-1	3576	O8:H7	*stx1*,* stx2*	*stx2*	*stx2*	*stx1 *(47%; 66%)	*stx2a*
PY222-3	2387	O185:H7	*stx1*	*stx1*	*stx1*		*stx1a*
PY233-4	58	O155:H21	*stx1*,* stx2*	*–*	*–*	*stx1 *(0%),* stx2 *(8%; 77%)	
PY233-5	8135	O116:H21	*stx1*,* stx2*	*stx1*,* stx2*	*stx1*,* stx2*		*stx1a*,* stx2a*
PY233-8	446	O22:H8	*stx2*	*stx2*	*stx2*		*stx2a*
PY233-9	446	O22:H8	*stx2*	*stx2*	*stx2*		*stx2a*
PY240-1	11729	O74:H42	*stx1*,* stx2*	*stx1*,* stx2*	*stx1*,* stx2*		*stx1a*,* stx2c*

^*a*^ Only subunit B was detected.

^*b*^ “–” indicates that the gene was not detected.

### Sub-typification of Shiga toxins and risk level classification

The sub-typification of Shiga toxins was performed on those samples in which the sequence of these genes was detected by WGS. Of the 13 isolates carrying the *stx1* gene, 11 belonged to the *stx1a* subtype, 1 belonged to the *stx1d* subgroup, and 1 sample could not be subtyped. Among the 22 isolates that carried the *stx2* gene, more than one subtype was found in 3 of them. The frequencies were 13 for *stx1a*, 3 for *stx2b*, 5 for *stx2c*, and 4 for *stx2d* subtype. Based on this sub-typification and according to the risk level classification suggested by FAO/WHO, 4 strains belonged to risk level 2 and 24 strains belonged to risk level 5.

### Serotypes, sequence types, and phylogroups

**Fig 1:**
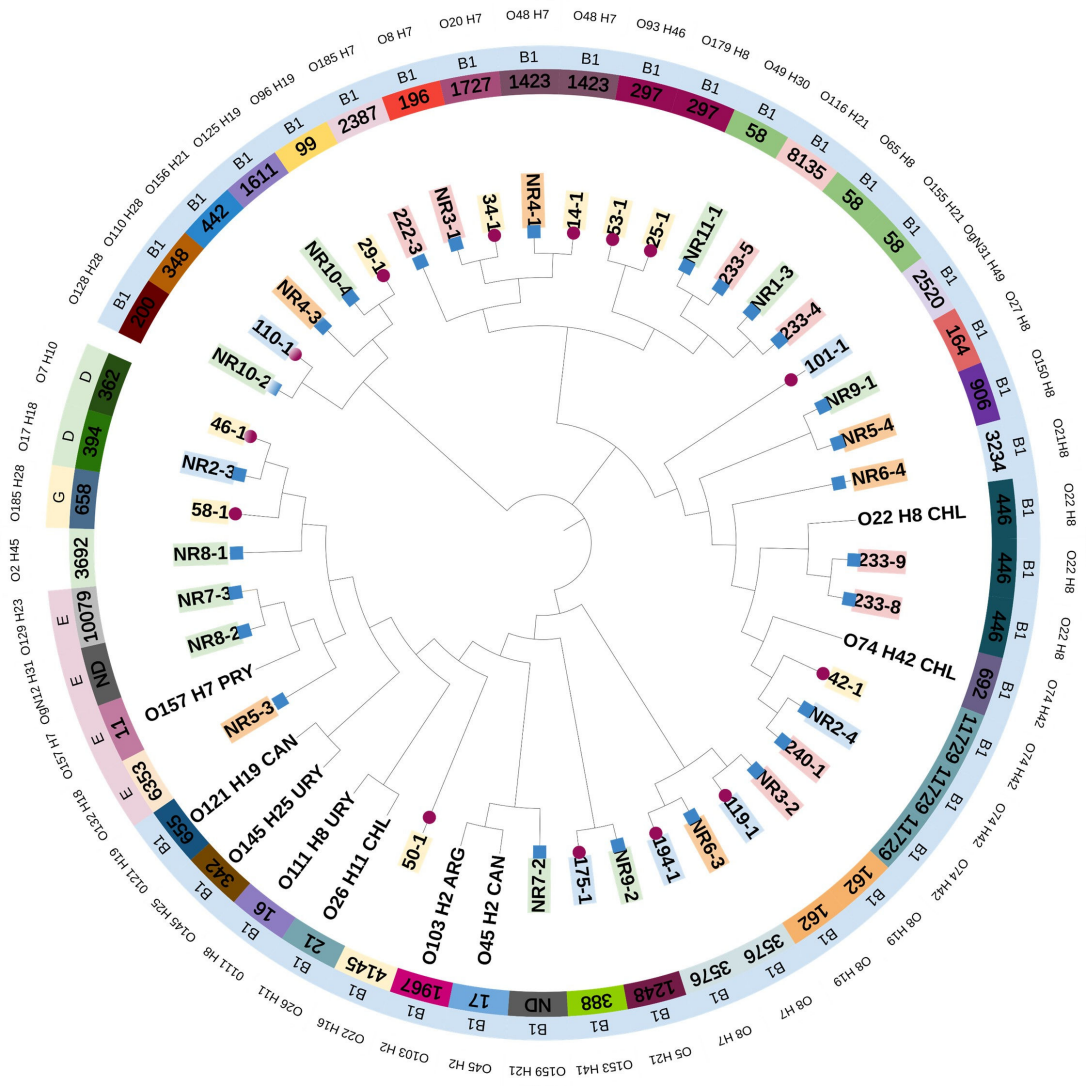
Phylogenetic and molecular characteristics of STEC isolated from Paraguayan cattle. Included are 38 strains characterized as STEC by conventional PCR, two strains not carrying toxins, and 13 sequences of STEC isolates collected in the Americas. The symbol at the end of the nodes represents the collection year of the specimen (blue square: 2016; pink circle: 2013). The color of the sample label indicates the department of Paraguay in which the sample was collected (green: San Pedro; orange: Caaguazú; yellow: Villa Hayes; blue: Paraguarí; rose: Cordillera). The inner circle indicates the different sequence types, the middle circle shows the phylogroups, and the outer circle indicates the samples' serotypes.

The analysis of the sequences allowed the detection of 34 different serotypes. The most frequently detected serotypes were O74:H42 (*n* = 3), O8:H7 (*n* = 3), O48:H7 (*n* = 2), and O22:H8 (*n* = 2). Two strains were classified as novel serogroups, OgN12 and OgN31. The serotypes of each of the analyzed strains are presented in Table 3 and Fig. 1.

**TABLE 3:** Characteristics of the strains obtained by WGS^*a*^

Strain	Year	O	ST	SG	PG	Res	Adhesion							Toxins		Siderophores			
							Adh	Fimbriae	Pili	Flagella	Curli fibers	Type II SS	Type III SS	Hemolysin	OT	Enterobactin	Ferrienterobactin	Ybt	Iron uptake
PY1-3	2016	SP	58	O65:H8	B1		*fdeC*	*fimABCDEFGHI*	*ecpABCDER*		*csgBDFG*	*gspCDEFGHIJKLM*	*espX*,* espL1*,* espR1*		*astA*	*entABCDEFS*	*fepABCDG*,* fes*		
PY2-3	2016	P	394	O17:H18	D		*fdeC*	*fimABCDEFGHI*	*ecpABCDER*		*csgBDFG*	*gspCDEFGHIJKLM*	*espX*,* espL1*, *e spR1*,* espL4*,* espP*,* espR4*,* espY*	*hlyABCD*		*entABCDEFS*	*fepABCDG*,* fes*		*chuSUVWY*
PY2-4	2016	P	11729	O74:H42	B1		*fdeC*	*fimABCDEFGHI*	*ecpABCDER*		*csgBDFG*	*gspCDEFGHIJKLM*	*espX*,* espL1*,* espR1*,* espP*	*hlyABCD*		*entABCDEFS*	*fepABCDG*,* fes*		
PY3-1	2016	Co	196	O8:H7	B1		*fdeC*	*fimABCDEFGHI*	*ecpABCDER*		*csgBDFG*	*gspCDEFGHIJKLM*	*espX*,* espL1*,* espR1*,* espP*	*hlyABCD*		*entABCDEFS*	*fepABCDG*,* fes*		
PY3-2	2016	Co	162	O8:H19	B1		*fdeC*	*fimABCDEFGHI*	*ecpABCDER*		*csgBDFG*	*gspCDEFGHIJKLM*	*espX*,* espL1*,* espR1*	*hlyABCD*		*entABCDEFS*	*fepABCDG*,* fes*		
PY4-1	2016	Ca	1423	O48:H7	B1	*fosA7*	*fdeC*	*fimABCDEFGHI*	*ecpABCDER*		*csgBDFG*	*gspCDEFGHIJKLM*	*espX*,* espL1*,* espR1*	*hlyABCD*	*cdtABC*	*entABCDEFS*	*fepABCDG*,* fes*		
PY4-3	2016	Ca	442	O156:H21	B1		*fdeC*	*fimABCDEFGHI*	*ecpABCDER*		*csgBDFG*	*gspCDEFGHIJKLM*	*espX*,* espL1*,* espR1*			*entABCDEFS*	*fepABCDG*,* fes*		
PY5-3	2016	Ca	6353	O132:H18	E		*fdeC*	*fimABCDEFGHI*	*ecpABCDER*		*csgBDFG*	*gspCDEFGHIJKLM*	*espX*,* espL1*,* espR1*,* espL4*,* espY*	*hlyABCD*		*entABCDEFS*	*fepABCDG*,* fes*		*chuUVWY*
PY5-4	2016	Ca	906	O150:H8	B1		*fdeC*	*fimABCDEFGHI*	*ecpABCDER*		*csgBDFG*	*gspCDEFGHIJKLM*	*espX*,* espL1*			*entABCDEFS*	*fepABCDG*,* fes*		
PY6-3	2016	Ca	3576	O8:H7	B1		*fdeC*	*fimABCDEFGHI*	*ecpABCDER*		*csgBDFG*	*gspCDEFGHIJKLM*	*espX*,* espL1*,* espR1*	*hlyABCD*		*entABCDEFS*	*fepABCDG*,* fes*		
PY6-4	2016	Ca	3234	O21:H8	B1	*fosA7*	*fdeC*	*fimABCDEFGHI*	*ecpABCDER*		*csgBDFG*	*gspCDEFGHIJKLM*	*espX*,* espL1*,* espR1*			*entABCDEFS*	*fepABCDG*,* fes*		
PY7-2	2016	SP	ND	O159:H21	B1		*fdeC*	*fimABCDEFGHI*	*ecpABCDER*		*csgBDFG*	*gspCDEFGHIJKLM*	*espX*,* espL1*,* espR1*			*entABCDEFS*	*fepABCDG*,* fes*		
PY7-3	2016	SP	10079	O129:H23	E		*fdeC*	*fimABCDEFGHI*	*ecpABCDER*		*csgBDFG*	*gspCDEFGHIJKLM*	*espX*,* espL1*,* espR1*,* espL4*,* espY*			*entABCDEFS*	*fepABCDG*,* fes*		*chuUVW*
PY8-1	2016	SP	3692	O2:H45	SD		*fdeC*	*fimABCDEFGHI*,* faeCDEFHIJ*	*ecpABCDER*	*fliN*	*csgBDFG*	*gspCDEFGHIJKLM*	*espX*,* espL1*,* espR1*,* espY*	*hlyABCD*	*astA*,* estla*	*entABCDEFS*	*fepABCDG*,* fes*		
PY8-2	2016	SP	ND	OgN12:H31	E		*fdeC*	*fimABCDEFGHI*,* f17d-CDG*	*ecpABCDER*		*csgBDFG*	*gspCDEFGHIJKLM*	*espX*,* espL1*,* espR1*,* espL4*,* espR4*,* espY*			*entABCDEFS*	*fepABCDG*,* fes*		*chuUVW*
PY9-1	2016	SP	164	O27:H8	B1		*fdeC*	*fimABCDEFGHI*,* papX*	*ecpABCDER*		*csgBDFG*	*gspCDEFGHIJKLM*	*espX*,* espL1*,* espR1*			*entABCDEFS*	*fepABCDG*,* fes*		
PY9-2	2016	SP	1248	O5:H21	B1		*fdeC*	*fimABCDEFGHI*	*ecpABCDER*		*csgBDFG*	*gspCDEFGHIJKLM*	*espX*,* espL1*,* espR1*			*entABCDEFS*	*fepABCDG*,* fes*		
PY10-2	2016	SP	200	O128:H28	B1		*fdeC*	*fimABCDEFGHI*	*ecpABCDER*		*csgBDFG*	*gspCDEFGHIJKLM*	*espX*,* espL1*,* espR1*		*astA*	*entABCDEFS*	*fepABCDG*,* fes*		
PY10-4	2016	SP	1611	O125:H19	B1		*fdeC*	*fimABCDEFGHI*,* faeEF*	*ecpABCDER*		*csgBDG*	*gspCDEFGHIJKLM*	*espX*,* espL1*,* espR1*			*entABCDEFS*	*fepABCDG*,* fes*		
PY11-1	2016	SP	58	O49:H30	B1		*fdeC*	*fimABCDEFGHI*,* f17d-CDG*	*ecpABCDER*		*csgBDFG*	*gspCDEFGHIJKLM*	*espX*,* espL1*,* espR1*,* espR4*		*astA*	*entABCDEFS*	*fepABCDG*,* fes*		
PY14-1	2013	VH	1423	O48:H7	B1	*fosA7*	*fdeC*	*fimABCDEFGHI*	*ecpABCDER*		*csgBDFG*	*gspCDEFGHIJKLM*	*espX*,* espL1*,* espR1*	*hlyABCD*	*cdtABC*	*entABCDEFS*	*fepABCDG*,* fes*		
PY25-1	2013	VH	297	O179:H8	B1		*fdeC*	*fimABCDEFGHI*	*ecpABCDER*		*csgBDFG*	*gspCDEFGHIJKLM*	*espX*, *espL1*, *espR1*,* espP*	*hlyABCD*	*cdtABC*	*entABCDEFS*	*fepABCDG*,* fes*		
PY29-1	2013	VH	99	O96:H19	B1		*fdeC*	*fimABCDEFGHI*	*ecpABCDER*		*csgBDFG*	*gspCDEFGHIJKLM*	*espX*,* espL1*,* espR1*,* espP*	*hlyABCD*		*entABCDEFS*	*fepABCDG*,* fes*		
PY34-1	2013	VH	1727	O20:H7	B1	*fosA7*	*fdeC*	*fimABCDEFGHI*	*ecpABCDER*		*csgBDFG*	*gspCDEFGHIJKLM*	*espX*,* espL1*,* espR1*			*entABCDEFS*	*fepABCDG*,* fes*		
PY42-1	2013	VH	11729	O74:H42	B1		*fdeC*	*fimABCDEFGHI*	*ecpABCDER*		*csgBDFG*	*gspCDEFGHIJKLM*	*espX*,* espL1*,* espR1*,* espP*	*hlyABCD*		*entABCDEFS*	*fepABCDG*,* fes*		
PY46-1	2013	VH	362	O7:H10	D		*fdeC*	*fimABCDEFGHI*,* afaABCDF*,* papX*	*ecpABCDER*		*csgBDFG*	*gspCDEFGHIJKLM*	*espX*,* espL1*,* espR1*,* espL4*,* espP*,* espR4*,* espY*	*hlyABCD*	*astA*,* cdtABC*,* cnf1*	*entABCDEFS*	*fepABCDG*,* fes*	*ybtAEPQSTUX*,* fyuA*	*chuUVW*,* irp1*,* irp2*
PY50-1	2013	VH	4145	O22:H16	B1		*fdeC*	*fimABCDEFGHI*	*ecpABCDER*		*csgBDFG*	*gspCDEFGHIJKLM*	*espX*,* espL1*,* espR1*,* espP*	*hlyABCD*		*entABCDEFS*	*fepABCDG*,* fes*		
PY53-1	2013	VH	297	O93:H46	B1		*fdeC*	*fimABCDEFGHI*	*ecpABCDER*		*csgBDFG*	*gspCDEFGHIJKLM*	*espX*, *espL1*, *espR1*,* espP*,* espR4*	*hlyABCD*		*entABCDEFS*	*fepABCDG*,* fes*	*ybtAEPQSTUX*,* fyuA*	*irp1*,* irp2*
PY58-1	2013	VH	658	O185:H28	G	*sitABCD*	*fdeC*	*fimABCDEFGHI*	*ecpABCDER*		*csgBDFG*	*gspCDEFGHIJKLM*	*espP*	*hlyABCD*		*entABCDEFS*	*fepABCDG*,* fes*		*chuTUVWXY*
PY101-1	2013	P	2520	OgN31:H49	B1		*fdeC*	*fimABCDEFGHI*	*ecpABCDER*		*csgBDFG*	*gspCDEFGHIJKLM*	*espX*,* espL1*,* espR1*,* espP*	*hlyABCD*		*entABCDEFS*	*fepABCDG*,* fes*		
PY110-1	2013	P	348	O110:H28	B1		*fdeC*	*fimABCDEFGHI*	*ecpABCDER*		*csgBDFG*	*gspCDEFGHIJKLM*	*espX*,* espL1*,* espR1*	*hlyABCD*		*entABCDEFS*	*fepABCDG*,* fes*		
PY119-1	2013	P	162	O8:H19	B1		*fdeC*	*fimABCDEFGHI*	*ecpABCDER*		*csgBDFG*	*gspCDEFGHIJKLM*	*espX*,* espL1*,* espR1*,* espP*	*hlyABCD*		*entABCDEFS*	*fepABCDG*,* fes*		
PY175-1	2013	P	388	O153:H41	B1		*fdeC*	*fimABCDEFGHI*	*ecpABCDER*		*csgBDFG*	*gspCDEFGHIJKLM*	*espX*,* espL1*,* espR1*	*hlyABCD*	*cdtABC*	*entABCDEFS*	*fepABCDG*,* fes*		
PY194-1	2013	P	3576	O8:H7	B1		*fdeC*	*fimABCDEFGHI*	*ecpABCDER*		*csgBDFG*	*gspCDEFGHIJKLM*	*espX*,* espL1*,* espR1*	*hlyABCD*		*entABCDEFS*	*fepABCDG*,* fes*		
PY222-3	2016	Co	2387	O185:H7	B1		*fdeC*	*fimABCDEFGHI*	*ecpABCDER*		*csgBDFG*	*gspCDEFGHIJKLM*	*espX*,* espL1*,* espR1*	*hlyABCD*		*entABCDEFS*	*fepABCDG*,* fes*		
PY233-4	2016	Co	58	O155:H21	B1		*fdeC*	*fimABCDEFGHI*,* afaA*,* faeCDEFHIJ*	*ecpABCDER*		*csgBDFG*	*gspCDEFGHIJKLM*	*espX*,* espL1*,* espR1*			*entABCDEFS*	*fepABCDG*,* fes*		
PY233-5	2016	Co	8135	O116:H21	B1		*fdeC*	*fimABCDEFGHI*	*ecpABCDER*		*csgBDFG*	*gspCDEFGHIJKLM*	*espX*,* espL1*,* espR1*,* espP*	*hlyABCD*	*cdtABC*	*entABCDEFS*	*fepABCDG*,* fes*		
PY233-8	2016	Co	446	O22:H8	B1		*fdeC*	*fimABCDEFGHI*	*ecpABCDER*		*csgBDFG*	*gspCDEFGHIJKLM*	*espX*,* espL1*,* espR1*			*entABCDEFS*	*fepABCDG*,* fes*		
PY233-9	2016	Co	446	O22:H8	B1		*fdeC*	*fimABCDEFGHI*	*ecpABCDER*		*csgBDFG*	*gspCDEFGHIJKLM*	*espX*,* espL1*,* espR1*			*entABCDEFS*	*fepABCDG*,* fes*		
PY240-1	2016	Co	11729	O74:H42	B1		*fdeC*	*fimABCDEFGHI*	*ecpABCDER*		*csgBDFG*	*gspCDEFGHIJKLM*	*espX*,* espL1*,* espR1*,* espP*	*hlyABCD*		*entABCDEFS*	*fepABCDG*,* fes*		

^*a*^ O, origin (SP, San Pedro; P, Paraguarí; Co, Cordillera; VH, Villa Hayes; Ca, Caaguazú); ST, sequence type; SG, serotype; PG, phylogroup; Adh, adhesion; SS, secretion system; OT, other toxins; Ybt, yersiniabactin.

Twenty-nine sequence types were identified among the isolates analyzed, with ST58 (*n* = 3) and ST11729 (*n* = 3) being the most frequent sequences. Two sequences could not be determined despite the seven genes showed agreement with the databases (Table 3).

Concerning the determination of phylogroups, 33 strains were classified as belonging to phylogroup B1; 3 strains belonged to phylogroup E, 2 strains to phylogroup D, and 1 isolated to phylogroup G. The phylogroup of an isolate could not be determined (Table 3; Fig. 1).

### Resistance and virulence genes

The presence of the *fosA7* gene, which confers resistance to fosfomycin, was detected in four isolates, and the *sitABCD* gene, which confers resistance to hydrogen peroxide, was detected in one isolate (Table 3).

**Fig 2:**
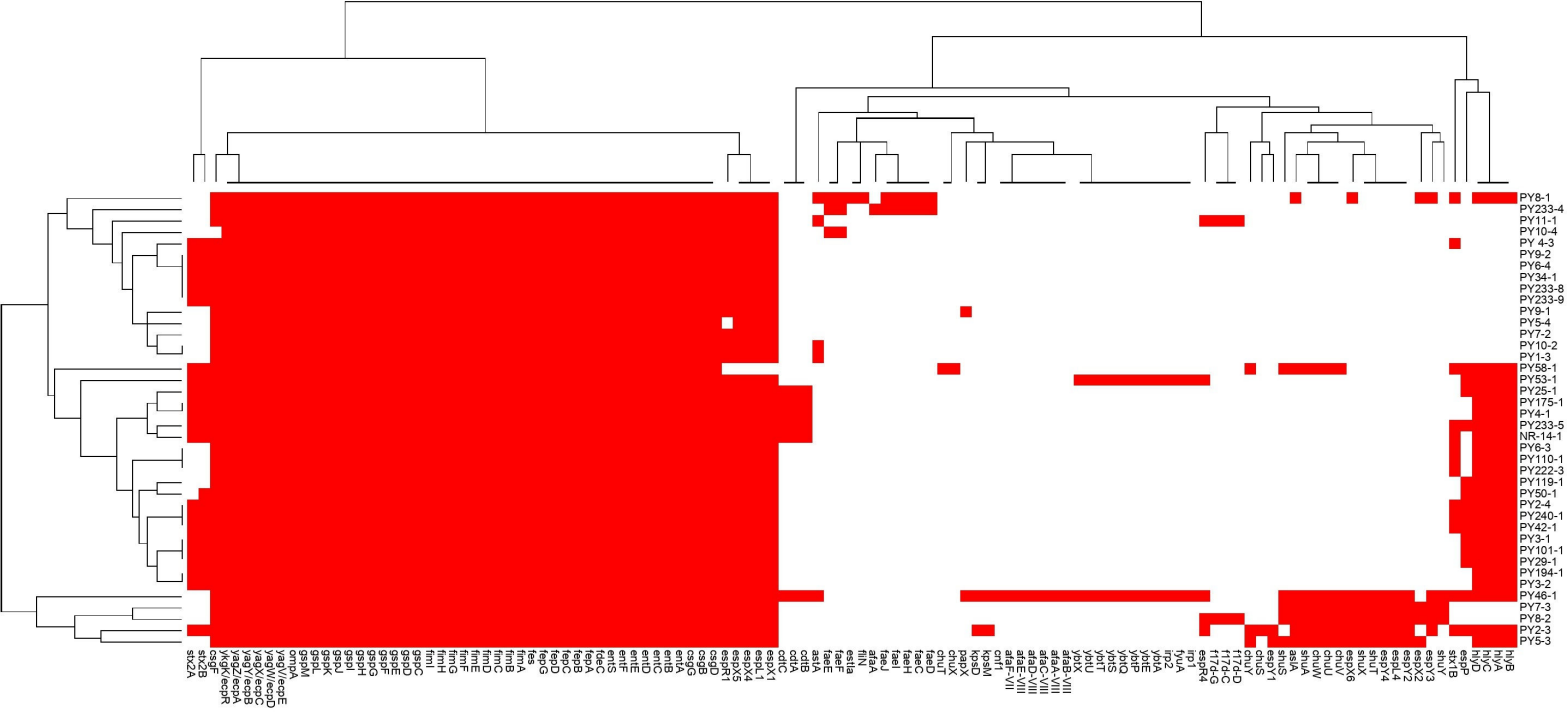
Virulence factors' heatmap and dendrogram from the *de novo* genome assembly of isolates of *E. coli* obtained from Paraguayan cattle (*n* = 40). Red boxes indicate the presence of each one of the virulence factors encoding the genes analyzed (*n* = 117). The isolates were hierarchically clustered based on their virulence factor profile using Euclidean metric distance with complete linkage clustering in both rows and columns, thus providing two dendrograms. The top dendrogram (top) clusters virulence factor according to their frequency in the isolates. The left dendrogram (left) clustered the isolates regarding their similarity in virulence profile.

Regarding virulence profiles, 27 different profiles were determined among the 40 isolates analyzed. The genes *csg* (*csgB*,* csgD*,* csgG*)*, ent *(*entA*,* entB*,* entC*,* entD*,* entE*,* entF*,* entS*)*, fdeC, fes, fep *(*fepA*,* fepB*,* fepC*,* fepD*,* fepG*)*, fim *(*fimA*,* fimB*,* fimC*,* fimD*,* fimE*,* fimF*,* fimG*,* fimH*,* fimI*)*, gsp *(*gspC*,* gspD*,* gspE*,* gspF*,* gspG*,* gspH*,* gspI*,* gspJ*,* gspK*,* gspL*,* gspM*), and *ecp *(*ecpA*,* ecpB*,* ecpC*,* ecpD*,* ecpE*,* ecpR*) were detected in all the isolates analyzed. Other genes detected with high frequency in the isolates were the *espX* (*espX1*,* espX4*,* espX5*) (*n* = 39), *espL1* (*n* = 39), *espR1* (*n* = 38), and *hly* (*hlyA*,* hlyB*,* hlyC*,* hlyD*) (*n* = 24). The comparison of the virulence profiles of the analyzed strains is presented in the heatmap in Fig. 2, and the complete list of genes detected in each isolated strain is presented in Table 3.

### Phylogeny

The core genome of our isolate collection included 2,907 genes. Reference sequences for serogroups corresponding to O157 and those in the group known as the “big six,” in addition to sequences of serogroups frequently found in this work, were included for better visualization of phylogenetic relationships. A clear clustering based on phylogroups can be observed in the tree. Despite the variability of sequences, it is also observed that isolates with the same sequence type belong to the same clade. However, there is a lack of phylogenetic relationships between the isolates concerning the year and place of collection, as seen in Fig. 1.

## DISCUSSION

To our knowledge, this is the first report describing complete genomes of STEC isolates collected from Paraguayan cattle. The high load of *stx* genes recorded by the initial screening shows the need to establish epidemiological controls, such as the one presented in this work, in search of strains that could represent a serious risk to human health.

For epidemiological surveillance, it is also important to compare the instruments and techniques used to carry it out. This work identifies discordances between PCR and WGS in detecting genes encoding Shiga toxins. It has been ruled out that this may be due to non-specific amplifications of the primers used by performing an *in silico* PCR, obtaining the same results as with the analysis of the WGS using the ABRicate tool. Additionally, we have dismissed the notion that the Ecoli_VF database’s inability to detect toxin genes is responsible. This conclusion is based on the additional comparison of reference sequences for the *stx1* and *stx2* genes against complete genomes, which consistently showed a coverage of 60% or less—below the 70% cut-off point established for the initial search with the database. The observed discrepancies between PCR and WGS in detecting Shiga toxin genes can be attributed to several factors. One significant reason is the loss of stx-encoding bacteriophages during subculturing processes. Bielaszewska et al. demonstrated that up to 14% of STEC colonies lost the *stx2* gene due to prophage excision during *in vitro* cultivation, leading to false-negative PCR results (32). It has been described that this loss can even occur with the first subculture (33, 34). Similar inconsistencies between PCR and WGS in the detection of *stx* genes have been previously reported. Castro et al. demonstrated that some isolates initially classified as STEC by PCR did not harbor complete *stx* genes when analyzed by WGS, but instead carried truncated remnants associated with defective prophages (35).

The fact that most of the isolates carried the *stx2* gene is alarming since this type of toxin is associated with more severe cases. Although studies have reported a similar prevalence of both toxins in STEC isolates associated with human infections, the difference observed in this study may be due to the animal origin of these isolates (36). Most of the isolates carrying *stx1* or *stx2* carried the *stx1a* or *stx2a* subtypes, which fits with other studies where a higher prevalence of these subtypes is reported both in isolates associated with infections in humans and isolates obtained from livestock. The *stx2a* subtype presents the highest risk of causing severe disease, in particular hemolytic uremic syndrome, characterized by acute kidney failure, and can affect other organs such as the lungs, pancreas, heart, and even lead to death (36, 37).

The WHO established a classification of potential risk of causing severe disease based on the presence of the *stx2a* type and subtype in conjunction with known adherence genes (*eae* or *aggR*), with level 1 being the highest risk and level 5 being the lowest risk. Four strains were classified as level 2, which represents a high risk of developing diarrhea, bloody diarrhea, and hemolytic uremic syndrome. Although most of the isolates were classified as level 5, it is important to consider that the severity of the symptoms also depends on other factors, such as those related to the host (12).

Determining the genetic characteristics of the strains and the phylogenetic analyses show significant variability among the analyzed strains without observing a predominance or a relationship between the serogroups and/or serotypes detected according to the region or year of collection.

There are specific serogroups associated with larger outbreaks and more severe conditions. This is the case with serogroups belonging to the group known as the “big six” (O26, O45, O103, O145, O111, and O121), in addition to serogroups O104 and O157. In the present study, none of the strains whose serogroup could be determined fall into any of these, which could be due to the fact that enrichment procedures for these serogroups were not performed. However, despite this, other factors should be considered to analyze the pathogenicity of these strains, such as virulence factors or their ability to persist on biotic or abiotic surfaces through biofilm (38).

Although none of the “big six” serogroups were recovered as isolates in our study, regional data suggest they are not absent from South America. For example, in Colombia, PCR detection from bovine fecal samples showed that the most recurrent serogroups were O45 (33.2%), O121 (23.8%), O103 (18.5%), O26 (12.1%), O145 (10.1%), and O111 (2.3%) (39). In Brazil, one O111 isolate was obtained from beef using PCR for the six major non-O157 serogroups, along with O8:H20, O22:H16, and O141:H49 (40). In Argentina, O145 has been detected in children with diarrhea (41), and retrospective studies found STEC O157 in 73.6% of patients with hemolytic uremic syndrome (HUS), O145 in 16.8%, O121 in 5.4%, and other serotypes in 4.2% (42). Importantly, some of the serotypes reported in this study have also been described in other countries in the region. Serotype O22:H8 was found in animal products in Argentina, while serotypes O74:H42, O185:H7, O8:H19, and O20:H7 were associated with strains isolated from bovine carcasses in Uruguay (43, 44).

Regarding the sequence types detected, ST58 was detected in three isolates in this study and has been recovered from different human and animal sources. Despite belonging to phylogroup B1, which is rarely pathogenic, the frequency of its isolation in bloodstream infections has increased in recent years (45). The ST162 sequence type, also described in this work, belongs to the pandemic clone isolated from various clinical and environmental sources (46, 47). At the same time, the ST3576 sequence has been isolated from fecal matter samples from healthy humans (48).

Most of the isolates (82.5%) belonged to phylogroup B1, which is consistent with the origin of these samples since this phylogroup is associated with animals and the environment, carrying genetic factors that allow its adaptation to soil, water, and even plants. Other phylogroups were also observed, albeit in low proportions, and correspond to phylogroups D, E, and G. Phylogroup D strains correspond mainly to extraintestinal pathogenic isolates in humans. In contrast, phylogroup E comprises isolates of various lifestyles (commensal, intraintestinal, and extraintestinal pathogen, environmental). On the other hand, the recently established phylogroup G can be associated with both animal and human isolates, although mainly with those related to extraintestinal pathologies (49–51).

The only antibiotic resistance gene detected was the *fosA7*, which encodes a glutathione S-transferase. This metalloenzyme gene can be transferred between bacteria of the family *Enterobacteriaceae* through plasmids and confers resistance to fosfomycin. However, the phenotypic determination of resistance to this antibiotic is necessary in addition to the genomic data (44, 52).

Regarding disinfectant resistance, an isolate carried the operon *sitABCD*, which encodes an ATP-dependent divalent metal ion transporter. The transport of manganese through this transporter contributes to the catalytic detoxification of reactive oxygen species, as this ion is a cofactor of several enzymes that contribute to protection against oxidative damage (53).

Among the virulence genes identified, those involved in bacterial adhesion to host cells and inter-bacterial interactions were the most frequently detected across the isolates. The *csg* genes participate in the synthesis of the curli protein, an amyloid-like fiber that constitutes the largest component of the protein portion of the biofilm in *E. coli* and take part in the colonization of surfaces (54). The operon *fim* houses the genes necessary to synthesize, assemble, and regulate type 1 fimbria. This fimbria plays a crucial role in the adhesion of bacterial cells to host cells, thus being a key component in biofilm formation and the bacterium’s survival (55, 56). The *ecp* genes encode a long filament known as *E. coli* common pilus, which also facilitates host adhesion and biofilm formation (57). A bacterial biofilm is a cellular conglomerate surrounded by an extracellular matrix of variable composition that confers resistance to bacteria against antimicrobial agents and the host’s immune system (58). Although the presence of these genes is indicative of the biofilm-forming capacity of these strains, phenotypic assays will be necessary to validate this finding given the complexity of the biofilm-formation process (59, 60).

The *ent*, *fes*, and *fep* genes are involved in the synthesis, transport, and functioning of enterobactin, a siderophore whose main function is to ensure the availability of the ferric ion required for bacterial metabolism (61–63). Other genes found in all the strains analyzed correspond to the gene *fdeC*, which encodes an intimin-like virulence factor that mediates adhesion to mammalian cells and extracellular matrix (64), and the *gsp* operon that encodes genes involved in the general secretory pathway. This secretion system mediates the excretion of proteins, including virulence factors, labeled with a signal peptide in a two-step process (65).

The *espX*, *espR*, and *espL* genes, also found with high frequency in these isolates, are effectors of the attaching and effacing (A/E) family that act through the type III secretion system that mediates their passage from the bacterium to the cytosol of the host cell (66). The operon *hlyABCD* contains genes that encode proteins necessary for the synthesis and transport of α hemolysin, which participates in the lysis of erythrocytes and other types of cells in the host (67).

Among the less frequent genes (present in 2–3 isolates), *espY1* and *chuY* were predominantly found in phylogroup D strains, often co-occurring with type III secretion systems and hemolysin operons. Phylogroup E strains exhibited distinct profiles involving *vat* and *irp* genes. These observations are consistent with prior research indicating that certain virulence factors are more prevalent in specific phylogenetic backgrounds (68).

These findings also raise relevant considerations from a food safety standpoint. Although none of the isolates belonged to high-risk serogroups, several combinations of virulence genes identified could represent a risk if introduced into the food production environment. The potential for biofilm formation and persistence suggests that these strains could survive on equipment or surfaces used during processing, increasing the chances of cross-contamination. It is also essential to consider these characteristics in light of the low tolerance that some international markets have for the presence of STEC in food products, which highlights the importance of involving next-generation sequencing tools in the epidemiological surveillance of this type of product, as they allow us to provide a broader overview of the characteristics of isolates that could represent a severe danger to human health.

Although this study focused on STEC isolates from cattle feces, we acknowledge the importance of evaluating additional points along the potential transmission chain to better understand how these strains may enter the food supply. Contamination can occur not only at the animal source, but also during slaughter, processing, or handling, through contact with surfaces, equipment, or workers. Our goal in this first phase was to describe the strains circulating at the animal level, as a starting point to understand their genetic profiles and potential risks. This is especially relevant in contexts like Paraguay, where limited WGS-based data is available, and the detection of genes related to biofilm formation, adhesion, and environmental persistence supports the need to study other stages in the production process in future work.

## Data Availability

All assemblies from this study are available at the NCBI Sequence Read Archive (BioProject PRJNA1127692).
